# Analysis of 605 tuberculosis outbreaks in Japan, 1993–2015: time, place and transmission site

**DOI:** 10.1017/S0950268821000625

**Published:** 2021-03-22

**Authors:** M. Ota, Y. Hoshino, S. Hirao

**Affiliations:** Research Institute of Tuberculosis, Tokyo, Japan

**Keywords:** Disease outbreak, health system strengthening, Japan, surveillance system, tuberculosis

## Abstract

Since 1993, reports on tuberculosis (TB) outbreaks have been collected in Japan; however, there has never been an overall analysis of these TB outbreaks. We aim to provide one here. The TB outbreak data were obtained from the Ministry of Health, Labour and Welfare and are described in terms of time, place and transmission site. The average number of TB cases and latent tuberculosis infection (LTBI) were compared by the transmission site. Some 605 TB outbreaks with 3491 TB cases were reported in 1993–2015 with an increasing trend (*r* = 0.45), during which time 728 777 TB cases were reported nationwide. On an average, TB outbreaks occurred more often in April to May (5.5 outbreaks per 2 months) than in December to January (3.4). The most common transmission sites were workplaces (*n* = 255), followed by health facilities (*n* = 144), schools (*n* = 60) and welfare facilities (*n* = 48). Psychiatric hospitals and nursing homes had the highest average number of TB cases per outbreak (8.5 each), whereas schools and prisons had the highest numbers of LTBI cases (29.1 and 38.9, respectively). Countries, particularly those that have resources to investigate TB outbreaks, should collect and analyse findings of TB outbreaks, as it informs surveillance systems and eventually strengthens general health systems.

## Introduction

In Japan, tuberculosis (TB) notification rate has declined about 60-fold in the past seven decades, from 698 per 100 000 population in 1951 to 12.3 per 100 000 population in 2018 [1]. However, about 5000 smear-positive TB cases are still reported annually [1] and these potentially infectious cases pose a public health threat to the community. TB outbreaks involving hospitals, including psychiatric hospitals, workplaces, the military, schools, prisons and sometimes the homeless, have also been reported [2–10].

Outbreak investigations of communicable diseases, including detection, prevention and response to outbreaks, are considered as an important part of the health systems [11], because they not only provide a scientific basis for implementing control measures to terminate the threats to the public's health but also opportunities for research about a problem and training of health personnel [12].

In 1993, the Ministry of Health and Welfare (MHW) of Japan started collecting reports from prefectures on ‘group infections’, defined as more than 20 cases of latent tuberculosis infection (LTBI), occurring in more than two households, and caused by one and the same source, modifying the old definition proposed by Drion *et al*. [13]. One case of TB disease is equivalent to six of LTBI in the definition. The Ministry has periodically provided brief feedback reports on the TB outbreaks; however, the feedback reports only included very brief analyses of time trends and transmission sites where the TB outbreaks often occurred [14]. This study aims to further epidemiologically describe the TB outbreaks reported to the Ministry from 1993 to 2015, in terms of time, place and transmission site, and compare the number of cases of TB disease and LTBI by site to explore the population at risk of TB outbreaks.

## Methods

A TB outbreak was arbitrarily defined in our study as an event in which three or more laboratory- or clinically diagnosed cases of TB disease were found in a well-defined setting such as a workplace, health or welfare facility, school, restaurant or household, to focus on medium to large TB outbreaks and to exclude small ones.

As noted in the introduction, in 1993 the MHW (currently the Ministry of Health, Labour and Welfare, MHLW) began to collect reports of TB outbreaks from prefectures and large cities all over Japan. A brief feedback from the MHLW is available on its website, which lists outbreaks that occurred from 1993 up to the end of 2015 [14]. The data were doubly entered in Microsoft Excel sheets and discrepancies were later resolved. The variables available were the time (year and month) when the index TB case was diagnosed, the prefectures and, in the outbreaks that occurred after 2002, the names of large cities (but not other administrative divisions), and the transmission sites in which the outbreaks occurred, as well as the numbers of cases of TB disease, LTBI, and persons under observation or follow-up. There were some outbreaks in which multiple sites involved in the outbreak were listed. When this was the case, two of the authors (MO and YH) individually and arbitrarily decided the main location of the outbreak, and then a third researcher (SH) decided the final site if there were discrepancies between the two initial reviewers. The 2005 census data for the whole of Japan and the prefectures were used for calculating the rates of TB outbreaks per 1 million persons.

The data were analysed in terms of time (yearly trend and seasonality), place (by prefecture and large city) and the transmission site. The seasonality was compared by month and consecutive 2-month periods. In comparing the occurrence of TB outbreaks by prefecture, the numbers of TB outbreaks per 1 million population in prefectures were calculated and compared. The numbers of TB outbreaks and the numbers of cases of TB disease and LTBI per transmission site were also calculated and compared among them.

R (Ver. x64 4.0.2, The R Foundation for Statistical Computing, Vienna, Austria) was used for all the statistical analyses, including calculating 95% confidence intervals. Pearson's correlation analysis was used for the trend of the yearly data. Student's *t* test was used for comparisons of the averages of two groups. Tukey's honestly significant difference test was used for multiple comparisons of data in groups. A *P* value of <0.05 was considered statistically significant.

An institutional ethical review was not necessary because this study utilised data that were already public and did not involve patients' identification or confidential information.

## Results

**Fig. 1.**
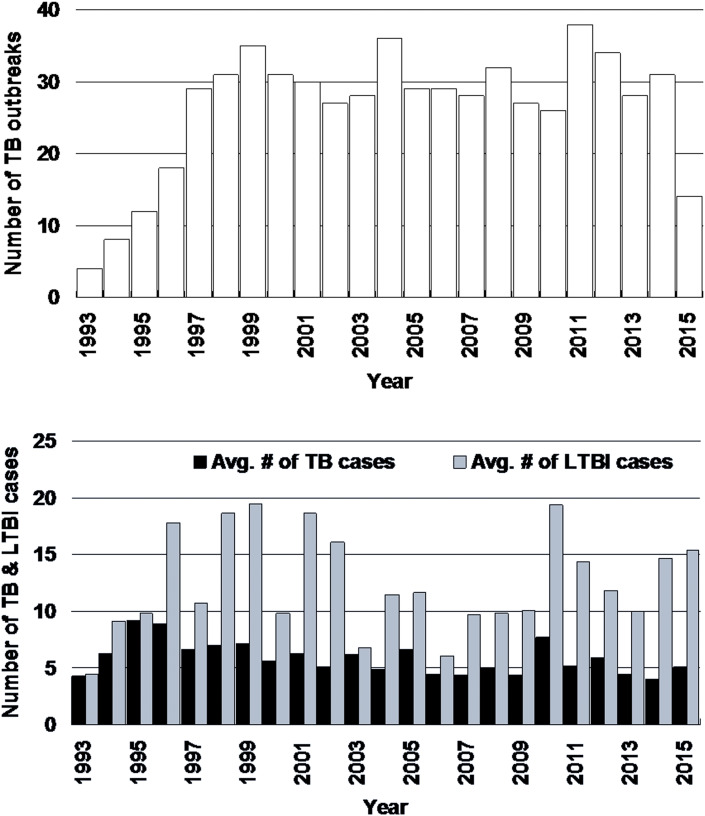
Long-term trend of tuberculosis outbreaks (upper) and the average numbers of cases of tuberculosis disease and latent tuberculosis infection (lower), Japan, 1993–2015 An increasing trend is exhibited (*r* = 0.45, 95% confidence interval: 0.05–0.73). # = number, AVG = average, TB = tuberculosis, LTBI = latent TB infection.

**Fig. 2.**
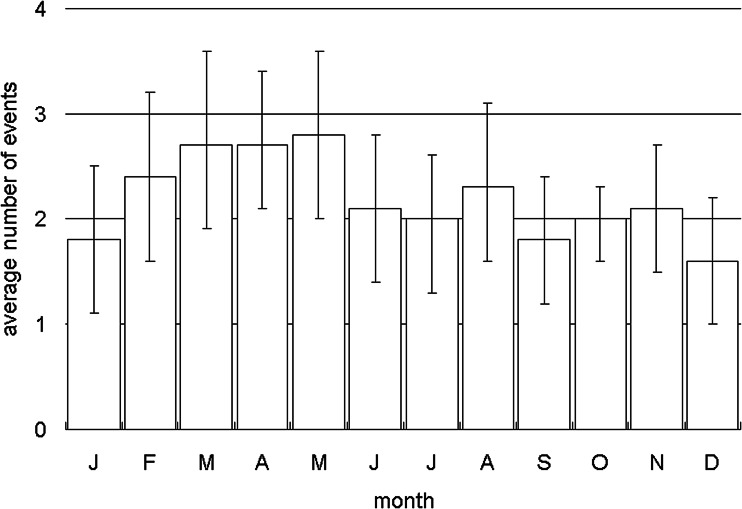
Distribution of tuberculosis outbreaks by month in Japan, 1993–2015. The month is defined as the one in which the index case of the tuberculosis outbreak was diagnosed. J = January, F = February, M = March, A = April, J = June, J = July, A = August, S = September, O = October, N = November, D = December. The error bars indicate 95% confidence intervals.

A total of 605 TB outbreaks with three or more cases of TB disease were found from 1993 through 2015. In 20 (3.0%) TB outbreaks, the two researchers' decisions on the main sites in which TB outbreaks occurred were different and the third researcher finally resolved the main sites. The average number of TB cases was 5.8 (95% confidence interval (CI): 5.3–6.2) per outbreak (range: 3–69), whereas the average number of LTBI cases was 12.8 (95% CI: 11.3–14.4) per outbreak (range: 0–152). The long-term trend of the outbreaks is shown in Figure 1 (upper). On average, 26.3 outbreaks (95% CI: 22.4–30.2) were reported per year and the number of reports exhibited an increasing trend with statistical significance (*r* = 0.45, 95% CI: 0.05–0.73). The average numbers of TB and LTBI cases per outbreak is shown in Figure 1 (lower). The average number of TB cases significantly decreased from 6.4 cases (95% CI: 5.7–7.1) per outbreak from 1993 to 2005, to 5.1 cases (95% CI: 4.6–5.6) per outbreak from 2006 to 2015 (*P* < 0.002). On the other hand, the number of LTBI cases did not significantly change (*P* = 0.28): it was 13.6 (95% CI: 11.1–16.1) per outbreak in 1993–2005, whereas it was 12.0 (95% CI: 10.3–13.6) per outbreak in 2006–2015. The average numbers of TB outbreaks that occurred in months in which the index TB patient was diagnosed are shown in Figure 2. The highest number (65) of TB outbreaks occurred in Mays (mean: 2.8, 95% CI: 2.0–3.6), whereas the lowest (37) in Decembers (mean: 1.6, 95% CI: 1.0–2.2). When compared by the consecutive 2-months period, the April–May period had the highest average number of TB outbreaks, with 5.5 (95% CI: 4.4–6.8) outbreaks per 2 months, whereas the December–January period the lowest, with 3.4 (95% CI: 2.3–4.5) outbreaks per 2 months and the difference was statistically significant (*P* < 0.05).

**Fig. 3.**
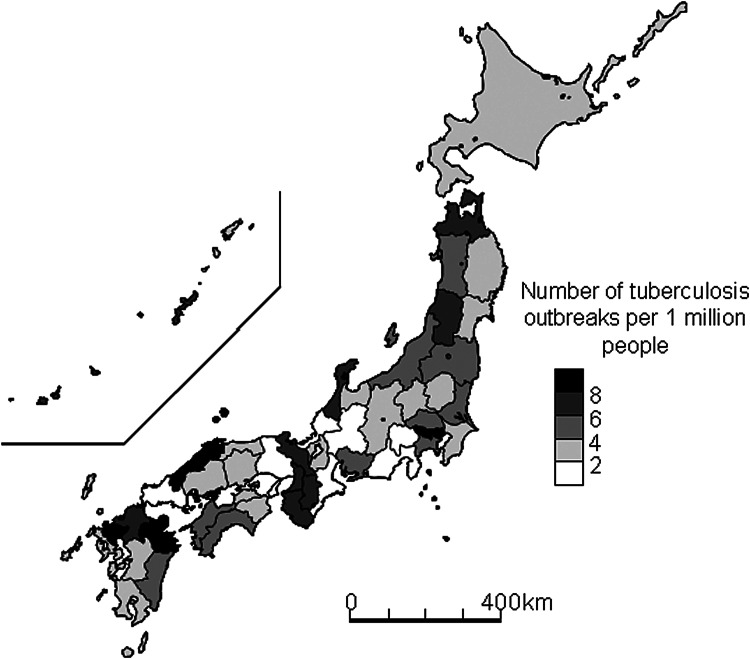
Geographic distribution of tuberculosis outbreaks per 1 million population in Japan by prefecture, 1993–2015.

Tokyo had 120 outbreaks, followed by Osaka (*n* = 62), Kanagawa (*n* = 37), Aichi (*n* = 35) and Fukuoka prefectures (*n* = 31). The fewest were in Fukui and Yamanashi (both: *n* = 0), Tottori, Tokushima and Yamaguchi prefectures (all: *n* = 2). After 2002, large cities where the outbreaks occurred were also specified in the list. The city of Osaka had 19 outbreaks, followed by Yokohama (*n* = 10), Nagoya and Fukuoka (both *n* = 9) and Kyoto and Kawasaki (both *n* = 7). The rate of TB outbreaks occurring per 1 million persons by prefecture is shown in Figure 3. The prefectures with the highest rates of TB outbreaks were mostly in western Japan, including Saga (10.4 per 1 million people), Shimane (9.4) and Oita (9.1), but some were also in eastern Japan, including Tokyo (9.5), and Yamagata prefectures (7.4). The prefectures with the lowest rates of TB outbreaks were Fukui and Yamanashi (both: 0.0), Yamaguchi (1.3), Gifu (1.4) and Shizuoka (1.6).

Table 1 shows the number of TB outbreaks that occurred and the average numbers of cases of TB disease and LTBI found by transmission site. Workplaces in general had the highest number of outbreaks (*n* = 253), followed by health facilities (*n* = 140), schools (*n* = 58) and welfare facilities (*n* = 49). Psychiatric hospitals had a significantly higher average number of TB cases (8.5 cases per outbreak, 95% CI: 6.7–10.3) than the workplaces (5.0 per outbreak, 95% CI: 4.6–5.4) and families (3.8 per outbreak, 95% CI: 3.4–4.2). The nursing homes also had a significantly higher average number of TB cases (8.5 cases per outbreak, 95% CI: 4.1–13.0) than families. In terms of LTBI cases, schools (29.0 per outbreak, 95% CI: 20.1–37.9) and prisons (38.9 per outbreak, 95% CI: 18.0–59.8) had higher average numbers than most of the other transmission sites. Of note, the average number of LTBI cases in schools decreased significantly from 35.1 (95% CI: 22.5–47.7) per outbreak in 1993–2005 to 16.5 (95% CI: 9.3–23.7) in 2006–2015. No other transmission sites exhibited a significant difference between the two periods (data not shown).

**Table 1.** The numbers of outbreaks and the average numbers of cases of tuberculosis disease and latent tuberculosis infection by transmission site in Japan, 1993–2015

		Tuberculosis disease				Latent tuberculosis infection				
Transmission site in which TB outbreak occurred	# of outbreaks	# of cases	mean	95% CIs		# of cases	mean	95% CIs	
Workplaces	253	1263	5.0	4.6	5.4^a^	2366	9.4	7.9	10.8^r^
Health facilities	140	988	7.1	6.2	7.9	1928	13.8	10.3	17.2
Hospitals/clinics	109	724	6.6	5.7	7.6	1545	14.2	10.1	17.8^gh^
Psychiatric hospitals	31	264	8.5	6.7	10.3^ab^	383	12.4	4.9	19.8^mo^
Schools	58	296	5.1	3.9	6.3	1682	29.0	20.1	37.9^fhjlopqr^
Welfare facilities	49	344	7.0	4.5	9.6	552	11.3	8.3	14.3
Welfare (excl. nursing homes)	27	156	5.8	2.7	8.8	306	11.3	7.6	15.1^q^
Nursing homes	22	188	8.5	4.1	13.0^c^	246	11.2	6.0	16.4^ij^
Family	30	114	3.8	3.4	4.2^bc^	118	3.9	2.7	5.2^def^
Restaurants	16	65	4.1	3.0	5.2	90	5.6	3.6	7.6^np^
Prisons	7	56	8.0	3.2	12.8	272	38.9	18.0	59.8^dgikmn^
Others	52	365	7.0	4.2	9.8	761	14.6	8.4	20.9^ekl^
Total	605	3491	5.8			7769	12.8		

avg = average, CI = confidence interval.

^a–r^The pairs the same superscript letters denote the pairs with statistically significant difference (*P* < 0.05).

## Discussion

We analysed 23 years of data on 605 TB outbreaks reported in Japan and found that the number of TB outbreaks increased in the last two decades, the index cases of the outbreaks were more often found from April to May than from December to January, and the outbreaks occurred more often per 1 million persons in western Japan. The average number of TB patients found per outbreak was highest in psychiatric hospitals and nursing homes, and the average number of LTBI patients found was highest in schools and prisons.

The number of outbreaks has been increasing, probably because as the incidence of TB decreases, TB cases may stand out more and TB outbreaks may more easily be detected [13]. Another reason would be that physicians and the public in general have become much less aware of TB and the diagnostic delay, as well as the delay in health seeking, has become longer, leading to increasing outbreaks. It is also possible that in early years of collecting the reports of TB outbreaks, particularly from 1993 to 1996, the local health staff members might not have been aware of the directive of the National TB Programme (NTP) and TB outbreaks might have been underreported. The index cases of the TB outbreaks were more often diagnosed in April to May than in December to January, probably because the 4 months preceding April to May (i.e. December to March) are in the winter when the average temperature is less than 10 °C in Japan [15], the windows of most buildings are shut to keep them warm inside, and ventilation is poor, facilitating TB transmission indoors [7, 16]. TB outbreaks occurred more often per 1 million population in western Japan, probably because the TB incidence is higher than average there [1]. Tokyo, the capital, had the fourth highest rate of outbreaks because the TB incidence is relatively high there [1]. However, another possibility is that occurrence of outbreaks reflects the quality or deficit of TB or communicable disease control practices of the prefecture, including delays in health-seeking of TB patients, diagnosis by physicians and contact investigations, as the top three prefectures in TB outbreaks per 1 million people were not the top three for TB notification rates. Osaka, Hyogo, and Tokyo (i.e. urban prefectures) were the regular top three prefectures for years [1]. The psychiatric hospitals involved in the outbreaks had more cases of TB disease than the workplaces in general, probably because in psychiatric hospitals in Japan, the patient's mental rather than physical condition is concentrated on and the delays of TB diagnosis tend to be longer [2, 17]. Another reason is that most psychiatric hospitals accommodate long-term inpatients and once a patient develops smear-positive TB in a ward other patients, and possibly staff members working in the same ward, may easily be exposed. Schools and the prisons had larger numbers of LTBI cases than most other settings, probably because these facilities have larger numbers of persons as contacts and a sputum smear-positive case can easily transmit TB bacilli to others nearby [6–8]. These facilities may also have an environment in which the air exchange rate is low, causing more TB transmission among contacts [7]. After 2006 when interferon-gamma release assays (IGRAs) [18–21] were introduced in Japan, the average number of TB disease per outbreak reduced significantly because it became easier for the health authorities to specifically determine those who were likely to be infected with TB and they may have been able to prevent them from developing TB disease via prophylaxis [17]. The introduction of IGRAs may also have resulted in a reduced number of cases of LTBI thereafter; however, it was only visible in the TB outbreaks in schools where most cases of LTBI found were children and young adults. This is because in late 2007 the criteria of LTBI treatment was revised to cover all the age-groups [22], whereas before 2007 only children and young adults up to 30 years of age were eligible for LTBI treatment [23], and the reduction of the number of the cases of LTBI enabled by the introduction of IGRAs was cancelled by the increased coverage of LTBI treatment.

There are a few review studies on TB outbreaks in the past. In the Netherlands, 44 TB outbreaks with more than six TB cases occurred from 1960 to 1964 [13]. They found a median of 6 to 10 TB cases per outbreak, which is similar to our findings, whereas the commonest transmission sites were different. They were family or close relations (*n* = 16), followed by ‘business and university’ (*n* = 10), and ‘schools’ (*n* = 8). In Cataluña, Spain, from 1998 to 2002, 27 TB outbreaks affecting 69 secondary cases of TB disease were reported (on average 3.6 TB cases per outbreak), of which the majority (*n* = 19) occurred within family members, six in the community and one each in school and the workplace [24]. The US Centers for Disease Control and Prevention (US-CDC) conducted two review studies on TB outbreaks for which the US-CDC provided epidemiological assistance [25, 26]. From 2002 to 2008, 27 TB outbreaks with more than three culture-confirmed TB cases occurred, with a median of ten cases of TB disease per outbreak [25]. The commonest transmission sites were ‘drug houses’ (*n* = 17), homeless shelters (*n* = 5), and correctional facilities (*n* = 4). From 2009 to 2015, 21 TB outbreaks occurred and the median number of cases of TB disease was nine. The commonest transmission sites were households (*n* = 9), overnight homeless facilities (*n* = 8), and local jails (*n* = 8)[26]. According to a review article on school TB outbreaks in China, 41% of the index patients were found in winter (December to February), followed by 28% in spring (March to May)[27], possibly because the Chinese study focused on school TB outbreaks.

There are some strengths and limitations to this study. The original data of this study were collected by the MHLW based on the directive of the NTP and the data are considered representative. Another strength is the large number of outbreaks included in the list without changing the reporting criteria for 23 years. On the other hand, one of the limitations would be that the study is based on secondary, brief data disclosed by the MHLW and the data are not comprehensive, limiting analysis on detailed factors such as the characteristics of TB in the cases. In addition, we had to rely exclusively on the classifications made by the MHLW, particularly the transmission sites. Second, the study was conducted in a country with a medium-burden of TB and the findings may not be generalisable to other countries. Third, since the outbreak reports from prefectures might not have been the final reports and the numbers of cases of TB disease and LTBI, not the final ones, the scope of the outbreaks might have been larger than described here. Even so, there were not any review reports on such a large number of TB outbreaks before and the authors believe it is of importance that this be published. Finally, the original data that MHLW disclosed did not clearly mention that the outbreaks were confirmed by molecular epidemiological evidence. However, the restriction fragment length polymorphism (RFLP) technique became available in Japan around 1995 and analysis of variable numbers tandem repeats (VNTR) became available around 2005 [28–32], so the authors believe that most outbreaks, particularly after 2000, were validated with the use of molecular epidemiology, exemplified by the TB outbreak reports [2, 4, 6, 8–10, 17].

There are several recommendations that can be made based on the findings of this and previous reports. As shown in our study, it is quite useful to collect and analyse the findings of TB outbreaks. Thus, countries, particularly those that have resources to investigate TB outbreaks, should collect and analyse the findings of such outbreaks, and share lessons learnt from them. We believe it will surely help TB surveillance systems to improve their capability to detect TB outbreaks, one of their major roles [33]. This would in addition strengthen general health systems in terms of prevention, detection and responses to disease outbreaks [11, 34]. Because disease outbreaks are ‘natural experiments’, they present opportunities to address questions germane to both basic and applied epidemiology [12, 35]. Since the introduction of IGRA for contact and outbreak investigations after 2006 seems to be reducing the number of TB cases per outbreak, the use of IGRA should be more widely adopted for such investigations, particularly in countries where BCG vaccination coverage is high and the results of tuberculin skin testing are likely to be confusing. Since psychiatric hospitals, nursing homes, schools and prisons had large numbers of cases of TB disease and LTBI when TB outbreaks occurred, they should strengthen TB screening for inpatients, clients, students, inmates and staff members with periodic chest X-ray examinations, if indicated. Since the capacity to respond to TB outbreaks varies, depending on the prefecture and city, the MHLW should build that capacity at the central level to provide technical assistance on-site such as TB mass screening, treatment and prophylaxis against MDR-TB, and epidemiology, as has been done in the US [36] and in Japan for acute communicable diseases [37]. The MHLW should also provide more detailed information on the outbreaks so that researchers can further analyse them and provide insights into TB outbreaks to the public health community.

## Data Availability

The data we used are open for the public in the URI indicated in reference 14.

## References

[1] Tuberculosis Surveillance Center, Research Institute of Tuberculosis. Tuberculosis in Japan. (2018) Annual Report. Available at https://www.jata.or.jp/rit/ekigaku/en/statistics-of-tb/ accessed on 19 August 2020.

[2] Ota M and Isshiki M (2004) An outbreak of tuberculosis in a long-term care unit of a mental hospital. (in Japanese) Kekkaku 79, 579–586. 15631110

[3] Seki N (2003) A suspected case of mass outbreak of tuberculosis infection in a small company separated into two floors. (in Japanese) Kekkaku 78, 395–399. 12806982

[4] Fujikawa A (2014) Tuberculosis contact investigation using interferon-gamma release assay with chest X-ray and computed tomography. PLoS One 9, e85612. 24454900 10.1371/journal.pone.0085612 PMC3891819

[5] Masuda M (2008) Usefulness of QuantiFERON TB-2G in a suspected case of drug resistant tuberculosis outbreak in a university. (in Japanese) Kekkaku 83, 7–11. 18283909

[6] Tasaka M (2018) A tuberculosis contact investigation involving a large number of contacts tested with interferon-gamma release assay at a nursing school: Kanagawa, Japan, 2012. Western Pacific Surveillance & Response Journal 9, 4–8. 10.5365/wpsar.2018.9.1.001 PMC6194223 30377544

[7] Matsumoto K (2011) An outbreak of tuberculosis in which environmental factors influenced tuberculosis infection. (in Japanese) Kekkaku 86, 487–491. 21735855

[8] Homma M and Itoh T (2014) Summary and issues of concern relating to a tuberculosis outbreak in a prison. (in Japanese) Kekkaku 89, 82–84.

[9] Kinoshita S (2007) Outbreaks of tuberculosis in facilities used by an unspecified number of people near a train station – problems regarding tuberculosis in urban areas. (in Japanese) Kekkaku 82, 749–757. 18018599

[10] Endo M (2019) A tuberculosis outbreak at an insecure, temporary housing facility, manga café, Tokyo, Japan, 2016–2017. Epidemiology & Infection 147, e222. 31364585 10.1017/S0950268819001092 PMC6625208

[11] Bloland P (2012) The role of public health institutetions in global health system strengthening efforts: the US CDC's perspective. PLoS Medicine 9, e1001199. 22509137 10.1371/journal.pmed.1001199 PMC3317896

[12] Goodman RA, Buehler JW and Koplan JP (1990) The epidemiologic field investigation: science and judgement in public health practice. American Journal of Epidemiology 132, 9–16. 2356818 10.1093/oxfordjournals.aje.a115647

[13] Drion R, Peters A and Kromsigt GJL (1968) Tuberculosis epidemic in the Netherlands. Bulletin of International Union Against Tuberculosis 41, 64–72. 5710263

[14] Ministry of Health, Labour, and Welfare (2019) List of tuberculosis outbreaks (in Japanese). Available at https://www.mhlw.go.jp/file/06-Seisakujouhou-10900000-Kenkoukyoku/0000148155.pdf (accessed 17 July 2020).

[15] Japan Meteorological Agency. Tables of climatological normals (1981–2010). Available at https://www.data.jma.go.jp/obd/stats/data/en/normal/normal.html accessed on 20 August 2020.

[16] Raffalli J, Sepkowitz KA and Armstrong D (1996) Community-based outbreaks of tuberculosis Archives of Internal Medicine. **156**, 1053–1060. 8638991

[17] Tasaka M (2020) A tuberculosis outbreak in a psychiatric hospital: Kanagawa, Japan, 2012. Epidemiology and Infection 148, 1–6. 10.1017/S0950268819002206 PMC7019127 31933448

[18] Ogiwara T (2013) Tuberculosis screening using a T-cell interferon-gamma release assay in Japanese medical school and non-Japanese international students. Tohoku Journal of Experimental Medicine. 230, 87–91. Available at: http://www.journal.med.tohoku.ac.jp/2302/230_87.pdf (accessed on 23 September 2020). 10.1620/tjem.230.87 23759899

[19] Higuchi K (2012) Comparison of specificities between two interferon-gamma release assays in Japan. International Journal of Tuberculosis & Lung Disease 16, 1190–1192. 10.5588/ijtld.11.0829 22748102

[20] Diel R, Loddenkemper R and Nienhaus A (2010) Evidence-based comparison of commercial interferon-gamma release assays for detecting active TB: a metaanalysis. Chest 137, 952–968. 20022968 10.1378/chest.09-2350

[21] Sester M (2011) Interferon-*γ* release assays for the diagnosis of active tuberculosis: a systematic review and meta-analysis. European Respiratory Journal 37, 100–111. 10.1183/09031936.00114810 20847080

[22] Yamagishi F (2005) Chemophylaxis. (in Japanese) Kekkaku 80, 647–653. 16296393

[23] Committees for Tuberculosis Prevention and Treatment, Japanese Society for Tuberculosis (2013) Guidelines for treating latent tuberculosis infection. (in Japanese) Kekkaku 88, 497–512.

[24] Bran CM (2006) Study of tuberculosis outbreaks reported in Cataluña (1998–2002). Archivos de Bronconeumologia 42, 260–266. 16827973

[25] Mitruka K (2011) Tuberculosis outbreak investigations in the United States, 2002–2008. Emerging Infectious Diseases 17, 425–431. 21392433 10.3201/eid1703.101550 PMC3166029

[26] Mindra G (2017) Tuberculosis outbreaks in the United States, 2009–2015. Public Health Reports 132, 157–163. doi: 10.1177/0033354916688270. 28147211 PMC5349481

[27] Bao H (2019) Tuberculosis outbreaks among students in mainland China: a systematic review and meta-analysis. BMC Infectious Diseases 19, 972. 31727001 10.1186/s12879-019-4573-3 PMC6854678

[28] Takahashi M (2002) Molecular epidemiology of Mycobacterium tuberculosis: its accomplishment and future perspective. (in Japanese) Kekkaku 77, 741–752. 12494513

[29] Takahashi M (2003) Molecular epidemiology of Mycobacterium tuberculosis using by RFLP analysis between genomic DNA. (in Japanese) Kekkaku 78, 641–651. 14621573

[30] Murase Y (2008) Promising loci of variable numbers of tandem repeats for typing Beijing family Mycobacterium tuberculosis. Journal of Medical Microbiology 57, 873–880. 18566146 10.1099/jmm.0.47564-0

[31] Hase A and Maeda H (2009) Progress of molecular epidemiology of Mycobacterium tuberculosis and its application for the prevention of tuberculosis. Kekkaku: [Tuberculosis] 84, 49–66.

[32] Matsumoto T and Iwamoto T (2009) Advances in molecular epidemiology of tuberculosis open the door to the next stage. Kekkaku: [Tuberculosis] 84, 783–794. 20077862

[33] Dato V, Wagner MM and Fapohunda A (2004) How outbreaks of infectious disease are detected: a review of surveillance systems and outbreak. Public Health Reports 119, 464–471. 15313109 10.1016/j.phr.2004.07.003 PMC1497658

[34] Hagan JE (2017) Use of a diagonal approach to health system strengthening and measles elimination after a large nationwide outbreak in Mongolia. Emerging Infectious Diseases 23(Suppl 1), S77–S84. 10.3201/eid2313.170594 PMC5711310 29155667

[35] Nsubuga P (2008) Training programme for field epidemiology. Lancet (London, England) 371, 630–631. 10.1016/S0140-6736(08)60281-0 18295009

[36] United States Centers for Disease Control and Prevention. Epidemiologic Assistance (Epi-Aids) Available at https://www.cdc.gov/eis/request-services/epiaids.html accessed on 20 August 2020.

[37] Field Epidemiology Training Programme, National Institute of Infectious Diseases. Field Epidemiology Training Programme. Available at https://www.niid.go.jp/niid/ja/fetp.html accessed on 20 August 2020.

